# Portfolio Effects and Synchrony in Coral Communities

**DOI:** 10.1002/ece3.73658

**Published:** 2026-05-14

**Authors:** Clinton B. Edwards, Jonathan A. Charendoff, Vid Petrovic, Stuart A. Sandin

**Affiliations:** ^1^ Scripps Institution of Oceanography University of California, San Diego La Jolla California USA; ^2^ Cooperative Institute for Marine and Atmospheric Research University of Hawaiʻi at Mānoa Honolulu Hawaii USA; ^3^ Pacific Islands Fisheries Science Center, National Marine Fisheries Service National Oceanic and Atmospheric Administration Honolulu Hawaii USA; ^4^ Computer Science and Engineering University of California, San Diego La Jolla California USA

**Keywords:** coral reefs, large‐area imaging, photogrammetry, portfolio effect, stability, synchrony

## Abstract

Theoretical and empirical work has demonstrated that population asynchrony can stabilize total community fluctuations through the portfolio effect. Stability and portfolio effects can arise as a statistical property of random fluctuations among populations in high diversity systems, or from predictable differences in biological responses to environmental fluctuations. Using a 9‐year coral abundance time series from 15 sites at a remote oceanic atoll, we determine if coral communities exhibit synchronous or asynchronous population dynamics, and whether these dynamics are driven by consistent patterns of covariation among populations. From 2013 to 2017 total community abundance was stable and associated with population asynchrony, driven by random fluctuations among populations, rather than predictable patterns of covariation among taxonomic groups. During the second phase of the study (2018 to 2021) community abundance declined dramatically and systematically, likely driven by a coral predator outbreak, and was associated with population synchrony. Coral populations were spatially and temporally variable in both phases of the study, and there was little support for patterns of either positive or negative covariation among taxonomic pairs as predicted by putative life‐history strategies. While the coral community demonstrated an inherent capacity for stability consistent with portfolio effects, this capacity was limited by the intensity and frequency of the disturbance regime.

## Graphical abstract

Reef‐building corals are well‐known for their taxonomic diversity and capacity for population variability. In investigating patterns of variability across coral taxa through a 9‐year annual time‐series, we found that a period of relative stability across the community was maintained through asynchronous fluctuations among constituent populations, suggesting statistically driven portfolio effects. Evidence of portfolio effects will not be ubiquitous, particularly when intense stressors homogenize species responses leading to coordinated loss and population synchrony across taxa.

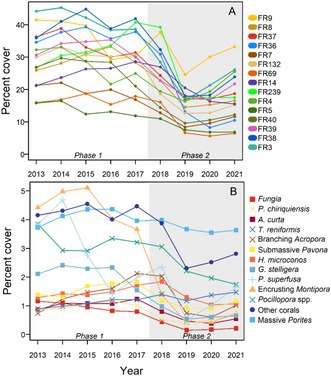

## 1 Introduction

The functional importance of species diversity is among the most studied and debated topics in ecology, with research in this area dating to the time of Darwin (Elton 1958; MacArthur and Levins 1967; Connell 1978; Hubbell 1997; Tilman 1999). Of particular interest is the relationship between species diversity and variability in total community abundance (MacArthur 1955; Tilman 1999; Zhao et al. 2022). Populations fluctuate according to intrinsic characteristics (Pianka 1970), interactions with other populations (Tilman 1999), stochastic events or, more generally, due to non‐stationary conditions (Rykaczewski and Checkley 2008). A substantial body of evidence has shown that when fluctuations between species are asynchronous, overall community stability can emerge (Tilman 1999; Wang et al. 2019; Zhao et al. 2022). Beyond theoretical applications, such views provide a practical framework for understanding and predicting how biodiversity scales to community dynamics (Hubbell and Foster 1992).

Uses of the term stability vary considerably, and often overlap with concepts of resilience, stasis, or resistance to perturbation or invasion (Elton 1958; Holling 1973; Tilman 1996). How fluctuations in the abundance of populations and communities contribute to stability has received considerable attention, particularly in recent years (Tilman 1999; Thibaut and Connolly 2013; Wang and Loreau 2016; Xu et al. 2021; Srednick et al. 2023). Stability, or lack thereof, can be represented at the species or community level, and across replicated temporal or spatial data series (Wilcox et al. 2017; Wang et al. 2019). Quantitatively, stability is often expressed as the inverse of the coefficient of variation in abundance (CV = *σ*/*μ*) (Tilman 1999; Lehman and Tilman 2000), and thus similarly expresses variability relative to the mean. Note, the inverse is often preferred over the CV itself as it takes larger values as data series become less variable or more stable. Regardless of how variability is presented, studies of stability have largely considered the relationship between species diversity and community variability, or lack thereof, with strong theoretical and empirical evidence for positive diversity‐stability relationships (Doak et al. 1998; Tilman et al. 1998; Cottingham et al. 2001; Xu et al. 2021).

When increased diversity is associated with asynchronous variability in individual populations, the consequence is reduced variability (i.e., increased stability) in measures of total community biomass or abundance (Tilman 1996; Xu et al. 2021). Various terms have been used to describe positive diversity‐stability relationships (DSR), including the portfolio effect, the insurance effect, or statistical averaging (Thibaut and Connolly 2013). These terms are related to the financial strategy of building diversified stock portfolios to minimize variability of investment returns (Markowitz 1999). The general theory of a positive DSR (e.g., the portfolio effect, sensu Thibaut and Connolly 2013) dates to the seminal works in the field of ecology (Elton 1958) and arose from observations of variation in species' response to disturbance and environmental forcings. There is strong empirical evidence for the portfolio effect, but debate remains regarding whether such effects typically can arise as a statistical property of increased diversity and random variability alone and without the need for any biotic mechanism (Doak et al. 1998; Tilman et al. 1998; Cottingham et al. 2001; Zhao et al. 2022). Importantly, the quantitative details of the portfolio effect are sensitive to patterns of covariation between the populations of individual species within the community, known as species synchrony.

The level of synchrony (or asynchrony) among populations in a community can be expressed as the ratio of total community variability to the sum of the variability of the individual populations, referred to as the synchrony index ([SI], Loreau and de Mazancourt 2008; Thibaut and Connolly 2013). When populations fluctuate synchronously, the SI takes a maximum value of 1; when there is no community variation, or when there is maximum asynchrony among populations, the SI equals 0. Total community variability is equal to the sum of the covariance matrix (Doak et al. 1998; Tilman 1999), and greater levels of negative covariation between populations will thus drive lower SI values. Negative covariation among taxa can arise from competition between species or from diverging demographic responses to short‐ or long‐term ecological change through an effect known as response diversity (Elmqvist et al. 2003; Gonzalez and Loreau 2009). Moreover, when declines in the abundance of some species are compensated for by gains in other species, overall community variability is reduced, even in low diversity systems (Cottingham et al. 2001; Downing et al. 2014; Brown et al. 2016).

Observations of tradeoffs in the traits of terrestrial plants and animals, and the consequence of these tradeoffs on demographic rates and measures of population size, form the basis of empirical and theoretical considerations of life‐history strategies (Pianka 1970; Grime 1977). Early studies of coral communities adapted life‐history theory frameworks to the study of coral reefs, expanding to incorporate the additional demographic flexibility of corals made possible by their modular life form (Jackson and Coates 1986). In addition to differing along familiar demographic axes, coral species differentiate in their ability to survive partial mortality, or proliferate following fragmentation (Highsmith 1982; Sandin, Eynaud, et al. 2020), thus providing an additional breadth of possible life‐history strategies. More recently, life‐history frameworks have been applied to understand community dynamics and describe diverging patterns of population change (Darling et al. 2012; Álvarez‐Noriega et al. 2016; McWilliam et al. 2020; Sandin, Eynaud, et al. 2020; McWilliam et al. 2023). Moreover, historical and contemporary shifts in coral assemblages (Loya et al. 2001; Montaggioni 2005; Pérez‐Rosales et al. 2021), suggest that asynchrony owing to the variety of available life‐histories may be characteristic of reef communities over longer time‐scales. However, the degree to which these classifications map onto realized demographic rates, or population‐ and community‐level fluctuations, remains ambiguous or unknown (Kuo et al. 2023; McWilliam et al. 2023). The study of corals at the population level has been complicated by the difficulty of replicating measurements underwater, particularly through time at fixed locations, which can obscure population‐ and site‐specific variability through time. As a result, investigations of the consequences of aggregate population fluctuations on overall coral community dynamics have been relatively limited.

There is some evidence that asynchrony between populations in different habitats can drive coral metapopulation stability (Edmunds and Smith 2022; Srednick et al. 2023). However, the degree to which coral communities display asynchrony to provide overall community stability, and how populations contribute to asynchrony within habitats remains unclear due to limitations in taxonomic‐ or plot‐level replication. Using a 9‐year time series of coral relative abundance from 15 plots at Palmyra atoll, we investigate patterns of covariation between coral taxa and look for evidence of portfolio effects. The study period featured a phase of relative stability with total community cover fluctuating around long‐term average, and a period of systematic decline associated with an outbreak of a coral predator, providing an opportunity to determine the ensemble of species responses driving both stability and decline in this diverse assemblage of corals. Finally, we test whether observed levels of synchrony are driven by consistent spatial patterns of covariance between species abundances, and whether these patterns align with theorized differences in life‐history strategies.

## 2 Methods

### 2.1 Study Location and Data Collection

Coral abundance data were collected at Palmyra Atoll from 2013 to 2021. Palmyra Atoll is a U.S. National Wildlife Refuge and is also part of the Pacific Remote Islands Heritage Marine National Monument. Located 1600 km south of Hawaii (Figure S1), the atoll is exposed to open ocean swells from the northern and southern hemispheres, including year‐round trade wind driven swells (Gove et al. 2015). A small research station with rotating staff and research teams is occupied year‐round, and aside from restoration of terrestrial habitats and a limited pelagic subsistence fishery, local human impacts to the atolls' marine ecosystems are extremely limited.

Large‐area imagery (LAI) was used to estimate coral proportional abundance inside permanent 10 m × 10 m study plots from 2013 to 2021 at 15 sites distributed along the 10‐m isobath fore reef (Figure S1, Edwards et al. 2017). The LAI approach uses overlapping images of natural scenes to create composite 3‐dimensional (3D) models and other derived 2‐dimensional products (i.e., orthoprojected maps) that are larger in extent than the constituent images from which they are formed (Edwards et al. 2023). For the purposes of this study, images were collected inside each plot, including an additional 1‐m buffer, with two high‐resolution (APS‐C or full‐frame sensor) cameras mounted to a single frame. The first camera uses a wide‐angle lens to provide the overlap among adjacent images required to build accurate 3D models, while the second camera uses a narrower field‐of‐view lens to capture the high‐resolution views needed for detailed taxonomic identifications. Imagery is collected by divers on SCUBA during a single 60–75 min dive per plot, with divers operating 1.5–2.5 m above the bottom to capture between 2000 and 4000 images per camera. Plots were visited annually in late summer or early fall, though due to delays resulting from the COVID‐19 pandemic, sampling was conducted during late fall in 2020 and 2021.

Raw images were processed into 3D models using the commercially available photogrammetric software Metashape Pro (Agisoft LLC, St. Petersburg, Russia). Output 3D models (i.e., dense point clouds) and camera position estimates were exported for use in the custom visual‐analytical software Viscore (Petrovic et al. 2014; Fox et al. 2019). Model scale and orientation with respect to the plane of the sea surface were applied in Viscore using four 50‐cm scale bars and depth information that was collected at six locations distributed throughout the plot during imaging. As time series for each plot became available they were spatially coregistered to the previous timepoints using manual and semi‐automated workflows in Viscore (Cook et al. 2023).

Based upon a shift in island‐wide environmental conditions, we a priori defined two phases of the time series. The first, 2013 to 2017, corresponded with observations of relative stability in estimates of percent coral cover, though also included the 2015–2016 warm‐water associated coral bleaching event. Accumulated heat stress at Palmyra reached 11.9 degree heating weeks before subsiding below alert levels by late December 2015. Despite significant coral bleaching and moderate impacts to the fore reef coral communities, mortality in adjacent habitats was low (Fox et al. 2019). The next phase of the study, 2018 to 2021, was defined by an outbreak of the Crown‐of‐Thorns starfish (COTS), a voracious coral predator (Pratchett et al. 2017), which occurred in elevated numbers on the fore reef for the first time since monitoring began at Palmyra. Observations of COTS, associated feeding scars, and impacts to the coral community were first visible during 2017 surveys (Table S1), lasting throughout the duration of the study, and until U.S. Fish and Wildlife led eradication efforts began in 2023.

### 2.2 Coral Abundance Estimation

The proportional abundance of corals was estimated as percent cover using the virtual point intercept (VPI) tool in Viscore (Fox et al. 2019). Within each plot a 10 m × 10 m sampling area was created and point samples (stratified random, 25 pts/m^2^) were generated from the top‐down perspective directly on to the 3D model. The same 10 m × 10 m area was used at each site across the coregistered time series, and a new set of points was generated for each year. Viscore was then used to interactively inspect the multiple raw images associated with the sampled points (Fox et al. 2019), and designate each to the finest possible taxonomic level. Corals were identified to the species level, or in groups comprised of 2–3 species but which tended to be dominated by a single abundant taxon. Some species (e.g., within *Acropora, Fungia* and *Montipora*) can be difficult to differentiate using imagery and were grouped to the genus or genus/morphology level. Details of the groupings used in this study, including prevailing life‐history strategy classifications, are provided in the Table S2. To provide additional ecological context, other benthic substrates (e.g., crustose coralline or fleshy algae, other invertebrates) were also classified.

To determine if island‐level changes in coral abundance were statistically significant across years we used repeated measures ANOVA. Since the data did not generally meet assumptions of normality or homoscedasticity, we used resampling to generate a bootstrapped null *F*‐distribution. Abundance values were resampled without replacement to randomly reassign group membership (i.e., year) and the *F*‐ratio was calculated. This process was repeated 10,000 times to create a null distribution of *F*‐ratios. Statistical significance was based on *α* = 0.05, and *p*‐values were generated by comparing the observed *F*‐ratios to the bootstrapped null distributions. Post hoc testing of group differences (i.e., between years) was also conducted via a bootstrapping procedure. Mean group differences were calculated using resampled group assignments (with replacement) and the process was repeated 10,000 times to create null distributions of group differences for each group pair. Observed differences were again compared to the bootstrapped distributions to generate *p*‐values.

### 2.3 Synchrony Calculations

Synchrony was calculated following the definition of the synchrony index (SI) as described in Thibaut and Connolly (2013):

(1) $$\varphi_{t}=\frac{\sum_{\mathrm{ij}}v_{n}^{s}\left(i,j\right)}{{\left(\sum_{i}\sqrt{v_{n}^{s}\left(i,i\right)}\right)}^{2}}$$

where $\sum_{\mathrm{ij}}v_{n}^{s}\left(i,j\right)$ is the summed temporal variance–covariance matrix, and $v_{n}^{s}\left(i,i\right)$ is the temporal population variance for each taxonomic group, i. The SI ranges between 1, when fluctuations in abundance are perfectly synchronous (i.e., positively correlated), and 0 when there is perfect asynchrony among population fluctuations, or when there is no fluctuation in total community abundance. The SI was calculated independently for using population abundances from each plot, and the island mean abundances, across two intervals: (1) before the observation of increased COTS abundance and feeding scars (2013 to 2017); and (2) after the observation of increased COTS (2018 to 2021).

### 2.4 Patterns of Synchrony and Covariation

To test whether observed synchrony values were reflective of communities composed of populations varying in a net positive or negative fashion, we created null distributions of synchrony values based on randomly varying coral group abundances (Vinebrooke et al. 2003). Abundance values were randomized for each coral group among years, and the new variance and covariance values were summed. This approach maintains constant levels of total community and population‐level variation in order to isolate the influence of covariation on synchrony values. We repeated this process 10,000 times to create a bootstrapped distribution of SI values for each site. Observed synchrony values below the 95% quantile range (QR) of the null distribution were considered significant evidence of negative covariation, while values greater than the 95% QR were considered evidence of positive covariation among populations.

A binomial test was then used to examine whether patterns of positive or negative covariance in the abundance of taxonomic pairs were consistent across sites. An equal probability of positive or negative covariance was assumed for all possible taxonomic pairs, $\left(i,j\right)$, and that the probability of positive or negative covariance was independent at each site. A binomial distribution was then used to calculate the probability of the observed number of positive or negative covariances given the total number of sites where each species pair occurred. Two‐tailed binomial probabilities of values equal to, or more extreme than, the observed values were summed to generate *p*‐values, and a threshold of *α* = 0.05 was used to determine statistical significance. All analyses were conducted using R version 4.1.2.

## 3 Results

### 3.1 Patterns of Total Coral Abundance

**FIGURE 1:**
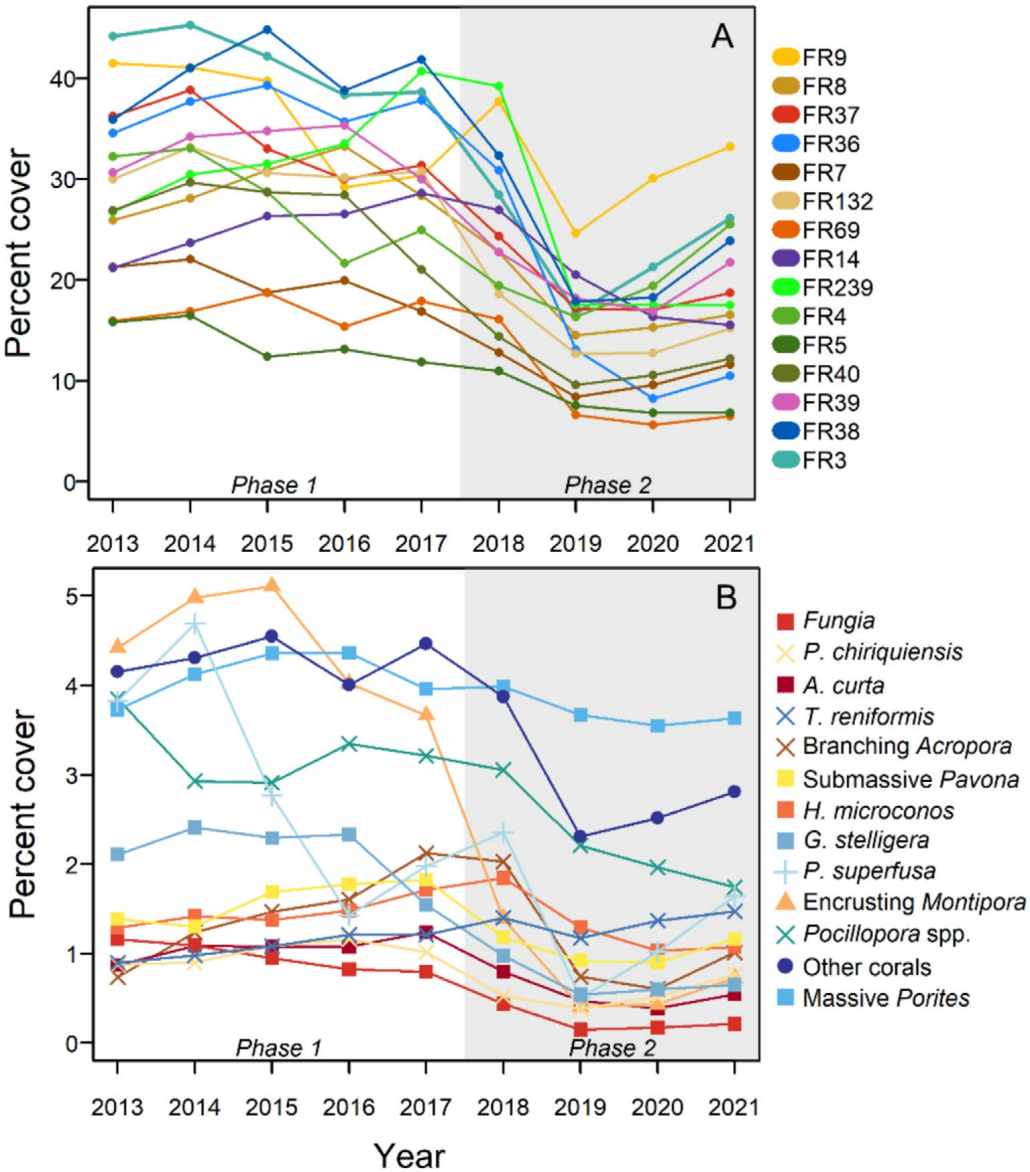
Total coral abundance (percent cover) at each site over the course of the study (A), and island mean yearly abundance (percent cover) of each focal coral group, including the sum of all other corals (B). Focal coral group names are organized by lowest to highest (top‐bottom) average overall abundance, and symbols represent the prevailing life‐history classifications: Stress‐tolerant (■), Competitive (×), Ruderal/Weedy (+), and Generalist (▲), following Darling et al. (2012). Unshaded and shaded regions of the plot correspond to the first and second phase of the study (2013 to 2017 and 2018 to 2021, respectively).

During the first phase of the study (2013 to 2017), there was no significant difference in mean coral cover (*F*_4,56_ = 2.23, *p* = 0.8). Notably, there was no change (*p* = 0.24) in mean coral cover between 2015 (30.7% ± 2.3 SE) and 2016 (28.6 ± 2.1 SE), the study interval which featured the warm water induced coral bleaching event. Phase 2 of the study was characterized by the sudden outbreak of COTS, which were first observed in 2017 with abundances as high as 7 ind./100 m^2^ in 2017 (Table S2), and continued to be observed at most sites throughout the course of the study. When COTS were first observed in 2017, mean coral cover was 28.7% (±2.3 SE), falling to 14.8% (±1.3 SE) by 2019, and remaining low thereafter (Figure 1A).

At the site level, the highest percent total coral cover was observed at FR3 in 2014, 45.3%, and the lowest cover at site FR69 in 2020, 5.6% (Figure 1A). Coral cover was more variable from site to site early in the time series, with the greatest range in 2015 (Mean: 30.7 ± 2.3 SE, Range: 12.4–44.9). By 2019, island‐wide declines in coral cover had reduced this variability (Mean: 14.8 ± 1.3 SE, Range: 6.6–24.6), though differential rates of recovery resulted in an increase in the variability of cover values by the end of the time series (Mean: 17.4 ± 1.8 SE, Range: 6.5–33.2). For all sites, the lowest observed total coral cover occurred either in 2019 (9 of 15 sites) or 2020 (6 of 15 sites). Cover values increased at most sites from 2020 to 2021 (13 of 15 sites); however, across the time series all sites experienced overall declines in total coral cover, with relative reductions in cover ranging from 20.0% (FR9, FR4) to 69.6% (FR36). While the two highest (FR9, FR3) and two lowest cover sites (FR5, FR69) remained as such by the end of the time series, the relative amount of change was variable for the remainder of the sites (Figure 1A).

### 3.2 Taxon Specific Patterns of Abundance

Across the time series, 34 coral groups were observed (Table S2), though most groups were found in low abundance. There were 12 focal coral groups identified as those whose island mean cover exceeded 0.5% during at least one year of the time series ( *Stylophora pistillata* also met this threshold, but was only present at 3 of the 15 sites and thus not included among the focal coral groups). Notably, the same 12 groups met a higher threshold of 1% island mean cover in at least one year, and on average represented 84.9% of the total coral cover across the time series (Range: 83.8%–86.4%). Of these 12 groups, 6 were identified to the species level, and 4 groups were comprised of 1–2 ecologically similar taxa. The remaining two groups, *Fungia* and encrusting *Montipora*, contain multiple species, but which are largely thought be functionally similar. All focal groups were present at each of the 15 sites during the time series, except for *Turbinaria reniformis* (12 sites), and branching *Acropora* (12 sites).

**FIGURE 2:**
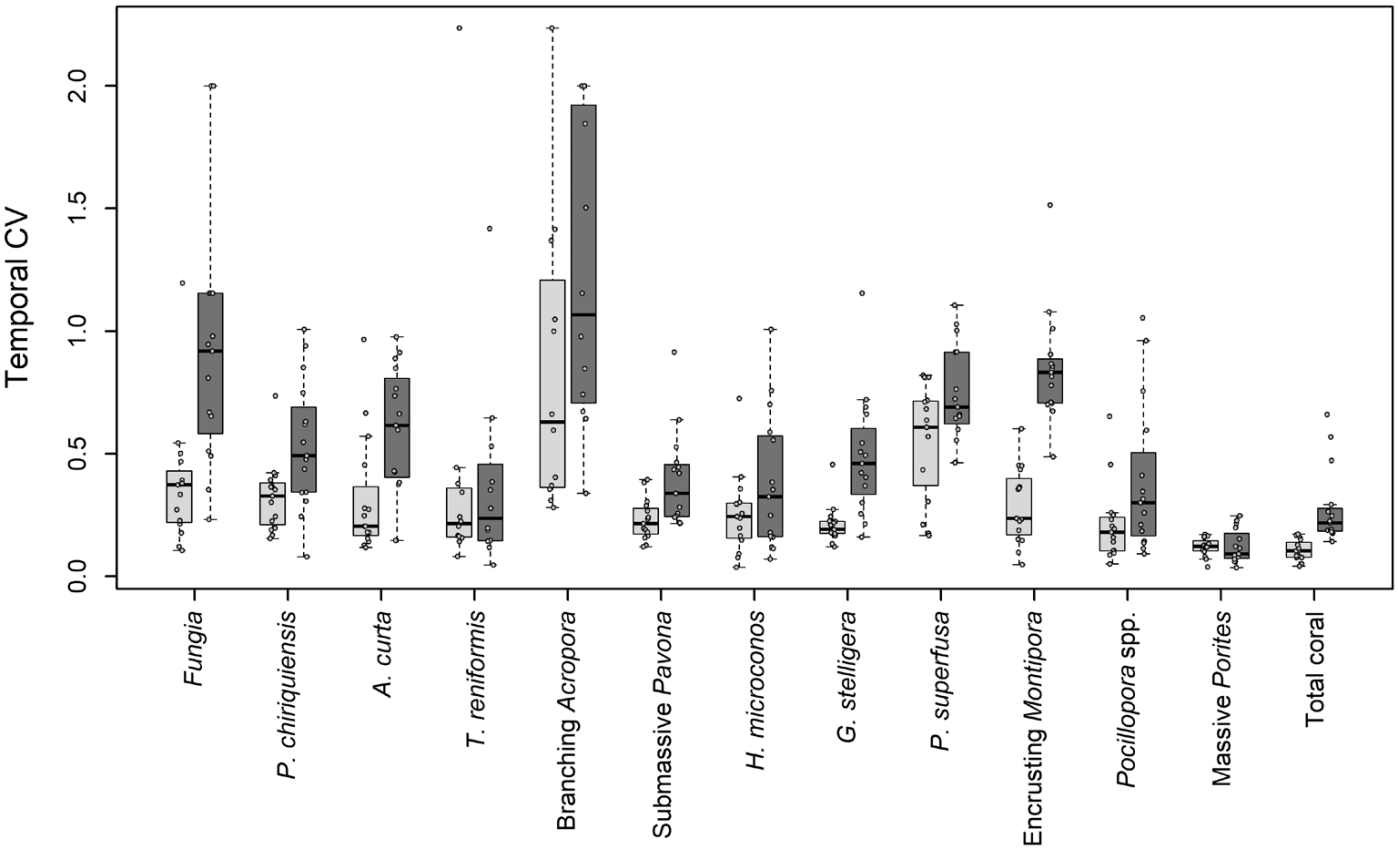
Boxplots of the coefficient of variation in abundance (CV = *σ*/*μ*) for each focal group, and total coral, in phase 1 (2013 to 2017, light‐gray) and phase 2 (2018 to 2021, dark‐gray) of the study, respectively. Groups are ordered by the average overall percent cover of each focal group (lowest‐highest, right‐left).

From 2013 to 2017, patterns of change were variable among focal groups, including little to no change in the abundance of most groups following the 2015 warm water event (Figure 1B). The largest declines during the 2015–2016 interval occurred in *Fungia*, encrusting *Montipora* and *P. superfusa*, while *Pocillopora* spp. abundance increased during this same time period. Following the COTS outbreak in 2017, all focal groups experienced declines in abundance, and the lowest abundance values for most focal groups occurred in 2019 or 2020 (11 of 13 groups; Figure 1B). From 2019 to 2021, most groups increased in cover, except for *Hydnophora microconos* , *Pocillopora* spp. and massive *Porites*, though this latter group remained the most abundant coral from 2019 to 2021 (Figure 1B). Moreover, while island level abundances generally decreased through time, there were considerable differences in temporal variability across sites for most focal groups (Figures 2 and S3). Site rank‐abundance was variable through time for all focal groups, though sites with the highest abundance were the same at the beginning and end of the timeseries for 9 of 12 focal groups (Figure S3).

### 3.3 Synchrony and Patterns of Covariation Among Coral Taxa

**FIGURE 3:**
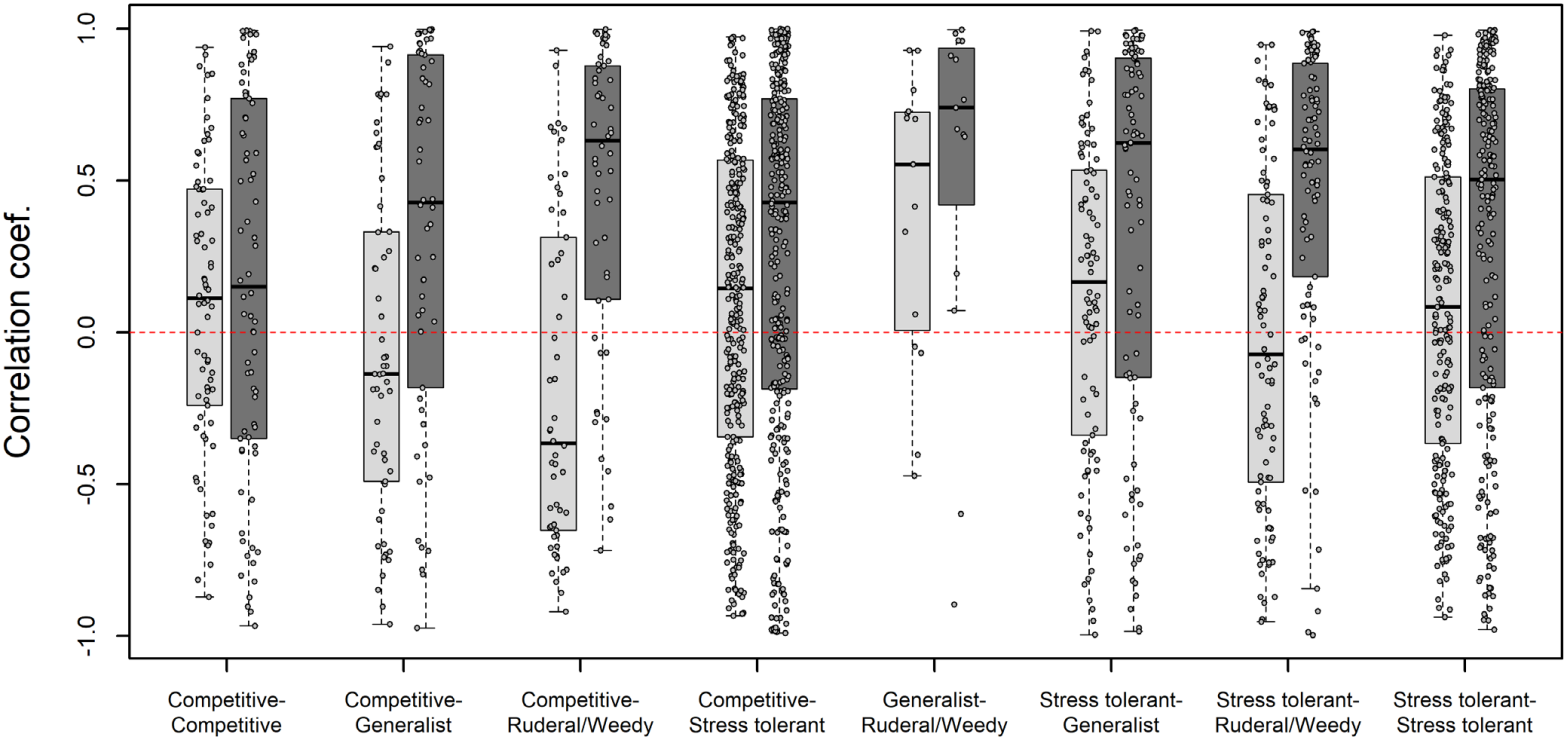
Boxplots of Pearson correlation coefficients (*r*) for abundance between all possible pairs of the 12 focal coral groups. Note, that while the numerator of the synchrony index includes covariation among taxonomic pairs (Equation 1), *r* is preferred for visualization as it scales between −1 and 1, making interpretation of the general increase in covariation from phase 1 to phase 2 more intuitive. Data are grouped by life‐history strategy pairings (i.e., Competitive vs. Competitive, Competitive vs. Generalist, etc.), individual points represent raw correlation values of site‐level abundance for specific species‐pairs within each life‐history category, and separated by phase. In Phase 1 (light‐gray boxes) the mean correlation coefficient across all focal group pairs was *r* = 0.06, and increased to *r* = 0.31 in Phase 2 (dark‐gray boxes).

Stability of coral cover in phase 1 of the time series was complemented by low SI values (0.03–0.41), reflecting a tendency for asynchronous fluctuations driven by weak levels of covariation among taxonomic pairs (Figure 3). However, observed SI values were not significantly different from distributions of randomly fluctuating populations (e.g., communities with no net positive or negative covariation among populations), except for sites FR132 and FR4 which had communities composed of net positively covarying species (Table 1). Conversely, in phase 2 of the time series observed SI values were consistently higher (0.15–0.89), and communities exhibited more positive covariation than expected by chance at 11 of 15 sites, as well as for the island mean (Table 1). The increase in the SI in phase 2 was associated with greater overall positive covariation among coral groups in phase 2 (Figure 3).

**TABLE 1:** Observed synchrony index values, with 95% quantile ranges (QR) from the bootstrapped null distributions for each site, and for the island mean abundances.

Site	Phase 1 (2013–2017)			Phase 2 (2018–2021)		
	SI	Null range	Significance	SI	Null range	Significance
FR132	0.27	0.01–0.25	+ covar	0.29	0.01–0.25	+ covar
FR14	0.18	0.01–0.19	NS	0.63	0.01–0.25	+ covar
FR239	0.25	0.03–0.27	NS	0.35	0.02–0.27	+ covar
FR3	0.05	0.01–0.21	NS	0.83	0.01–0.31	+ covar
FR36	0.15	0.02–0.24	NS	0.36	0.01–0.24	+ covar
FR37	0.03	0.01–0.19	NS	0.30	0.01–0.23	+ covar
FR38	0.09	0.03–0.29	NS	0.88	0.04–0.43	+ covar
FR39	0.22	0.01–0.24	NS	0.61	0.01–0.32	+ covar
FR4	0.41	0.01–0.22	+ covar	0.77	0.04–0.41	+ covar
FR40	0.22	0.06–0.35	NS	0.31	0.03–0.35	NS
FR5	0.26	0.04–0.29	NS	0.55	0.02–0.42	+ covar
FR69	0.1	0.02–0.25	NS	0.18	0.01–0.21	NS
FR7	0.06	0.01–0.22	NS	0.15	0.01–0.27	NS
FR8	0.06	0.03–0.32	NS	0.47	0.19–0.5	NS
FR9	0.09	0.02–0.27	NS	0.30	0.01–0.22	+ covar
*Island mean*	0.06	0.02–0.24	NS	0.74	0.01–0.24	+ covar

*Note:* Observed SI values outside of the 95% QR's of the null distributions are indicative of communities comprised of significantly positively (+ covar) or negatively (− covar) covarying species, the latter which was not observed in either phase of the study. Observed SI values within the 95% QR's of the null distributions were not significantly different (NS) from communities comprised of randomly fluctuating species.

**FIGURE 4:**
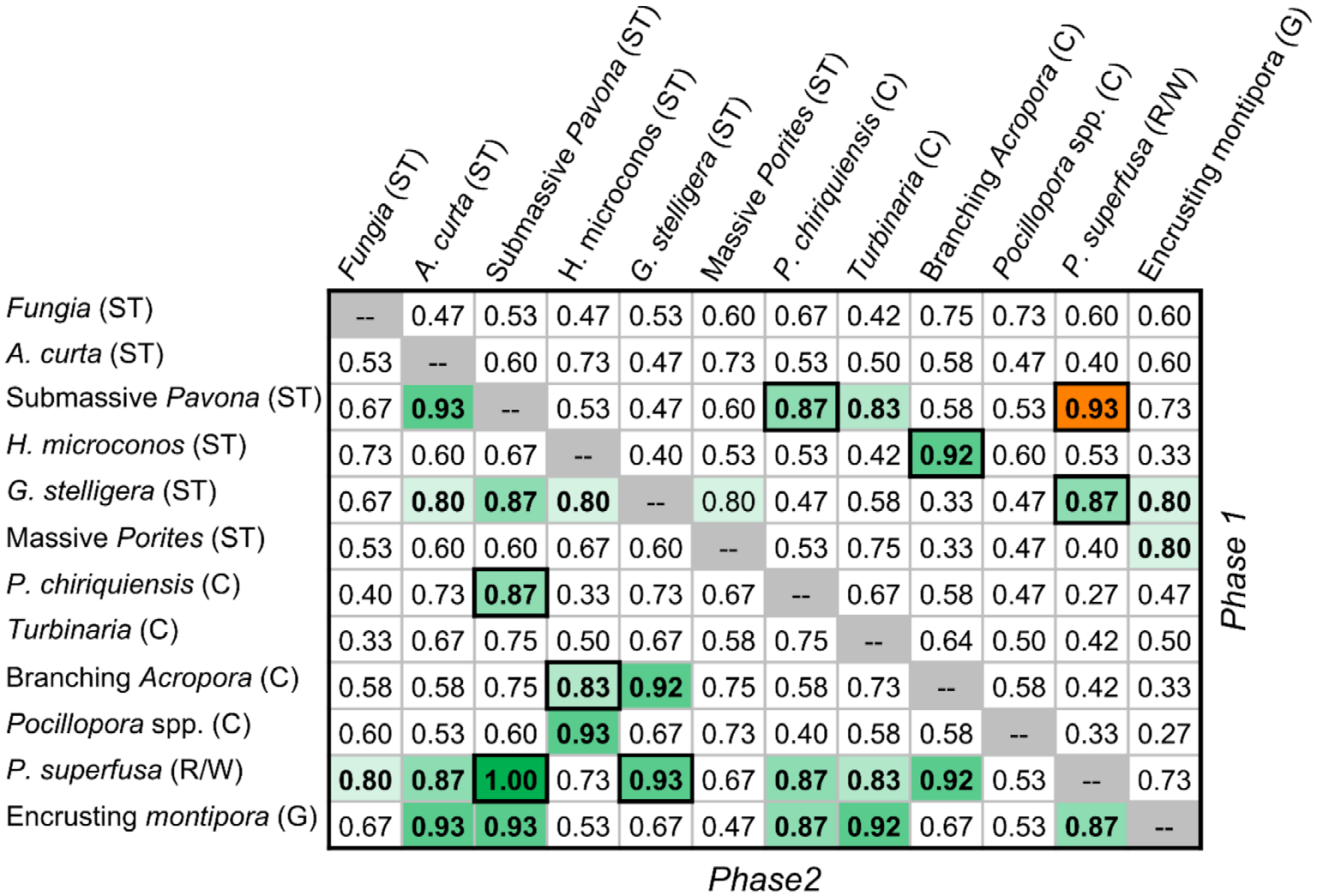
Proportion of sites with positive or negative covariance in phase 1 (2013 to 2017, upper triangle) and phase 2 (2018 to 2021, lower triangle) for focal group pairs. Focal groups are grouped by prevailing life‐history classification (C = Competitive; ST = Stress tolerant; R/W = Ruderal/Weedy) following Darling et al. (2012), and ordered from highest to lowest overall mean abundance within groupings. Highlighted cells and bold text indicate statistically significant (α = 0.05) proportion of sites with similar covariation (e.g., positive or negative), with darker colors corresponding to higher proportions. Cells outlined in bold indicate species pairs with significant patterns of covariation in both phases of the study. Only one species pair demonstrated significant negative covariation (*P. superfusa* and submassive *Pavona*) in either phase of the study.

Temporal covariation was calculated for each of the 66 possible focal group pairs at each site. All pairs occurred in at least 12 of 15 sites in both phases of the time series, and 45 pairs occurred at all 15 sites in both phases. In phase 1, 8 of the 66 focal group pairs positively covaried more frequently at the site level than expected by chance, and the only pair to consistently negatively covary across sites was *P. superfusa* and submassive *Pavona* (Figure 4). Conversely, in phase 2 community wide declines in abundance led to more consistent patterns of covariation, and 20 focal group pairs were found to consistently positively covary across sites, including *P. superfusa* and submassive *Pavona* which had previously negatively covaried. Only four focal pairs had significant covariance patterns in both phases of the time series (Figure 4).

## 4 Discussion

Over the course of the 9‐year study we saw contrasting patterns of community stability in response to disturbance. During the first phase of the study (2013 to 2017) asynchrony in population fluctuations led to stability in total community abundance, providing evidence for portfolio effects in this phase of the time series. We did not find support for consistent patterns of negative covariation among taxonomic pairs as a factor contributing to asynchrony. These results suggest that population stability and portfolio effects in this phase are more likely to have emerged due to demographic stochasticity driven by local environmental and ecological conditions rather than response diversity associated with predictable differences in life‐history strategies among taxa. The second phase (2018 to 2021) of the study was characterized by the outbreak of COTS, the effects of which were felt across the coral community. Likely due to the effects of multi‐species mortality, we found consistent evidence for increased positive covariation between taxonomic pairs (Figure 3) driving higher levels of synchrony in the period from 2018 to 2021. With few exceptions, patterns of abundance and covariation did not fit expectations of putative differences in life‐history strategy (e.g., competitive taxa did not suffer greater reductions in abundance relative to stress‐tolerant taxa; Figures 4 and S3). Further, species and community level patterns of abundance and covariation were highly variable from site to site (Figures 4 and S2), suggesting that local ecological and environmental context remains of first‐order importance to understanding local population status at any given point in time.

Theoretical and empirical work provides strong evidence that stability in high diversity systems (e.g., portfolio effects) can arise from random demographic variation alone (Doak et al. 1998; Tilman et al. 1998; Zhao et al. 2022), but that such effects can also be driven by variation in ecological strategy and response to environmental forcings (Gonzalez and Loreau 2009; Brown et al. 2016). Broadly known as response diversity, varying or contrasting patterns of change among species should lead to strong patterns covariation (both positive and negative) among populations that can enhance community stability, even in relatively low diversity systems (Doak et al. 1998; Gonzalez and Loreau 2009; Brown et al. 2016). In the first phase of the study, low SI values were associated with stability in total community abundance (Table S3), despite variation among populations (Figure 2). However, in phase 1 the observed synchrony values did not differ from those generated by simulated communities of randomly fluctuating species (Table 1). These results are consistent with portfolio effects driven by random demographic variation, rather than response diversity. Moreover, our results suggest that any differences in life‐history strategies among corals were not of sufficient magnitude to lead to consistent patterns of negative (or positive) covariation among populations. Previous work has suggested that demographic stochasticity can drive community stabilization and obscure phenomena such as compensatory dynamics in some settings (De Mazancourt et al. 2013), though such effects are unlikely here given the consistency of our findings across sites. Further, it has been suggested that compensatory dynamics require strong interactions or competitive release to occur (Brown et al. 2016). However, corals are not known to have strong direct interactions in natural settings (Álvarez‐Noriega et al. 2018) and, nor is there evidence for space preemption by other non‐coral taxa in our data. For instance, the coverage of crustose coralline algae, which is competitively inferior to coral (Price 2010; Buenau et al. 2011), averaged from 23.2% to 30.0% over the course of this study (Figure S4), suggesting coral growth was not space limited. Regardless, given the demographic flexibility available to corals, it is possible that patterns of covariation along particular demographic axes, such as rates of shrinkage or regrowth, were not captured with the bulk metric of proportional abundance, highlighting the need for more detailed and spatially explicit demographic investigations of coral populations.

The second phase of the study (2018 to 2021) was characterized by a dramatic decline in community abundance and stability (Figure 1 and Table S3) associated with greater SI values driven by increased positive covariation between populations within and across sites (Table 1 and Figure 3). Previous work has shown that variability in population responses to heterogeneous environmental conditions can enhance asynchronous dynamics (Hallett et al. 2014; Wang et al. 2019). Conversely, widespread and high‐intensity forcings can homogenize these responses, leading to declines in community abundance and diversity in the long term, or large‐scale systematic shifts in community structure (Sandin, Eynaud, et al. 2020). While the first phase of the study included a significant heat stress event, the impacts of this disturbance were relatively brief and most coral populations were largely able resist mortality, or rapidly recovered (Figure 1B; Fox et al. 2019). On the other hand, elevated COTS densities were first observed in 2017 and within two years total coral cover had declined by nearly 50% (Figure 1A), with the effects felt ubiquitously across the coral community (Figure 1B). Other drivers of increased synchrony, including species richness and evenness (Thibaut and Connolly 2013) were unchanged through time, or variable from site to site (Figure S5), and are unlikely to have contributed to the increased synchrony observed at all sites. While we failed to detect response diversity and stability on the fore reef during the second phase of this study, differences in environmental gradients and community responses in adjacent habitats (such as the vast western terrace where COTS have not been reported) may help to provide overall metapopulation stability on Palmyra (Wilcox et al. 2017; Srednick et al. 2023). Overall, the increased levels of synchrony and positive covariation observed here are consistent with a lack of response diversity but are also likely due to the high level of disturbance in the second phase of the study.

Life‐history theory offers an appealing framework for describing coral community dynamics and has been increasingly applied to coral communities (Darling et al. 2012; Kuo et al. 2023; McWilliam et al. 2023). Evidence from contemporary and historical assemblage shifts provides strong evidence for changes in coral communities resulting from differential response of species to environmental fluctuations (Loya et al. 2001; Montaggioni 2005; Darling et al. 2013; Pérez‐Rosales et al. 2021). However, the lack of strong patterns of covariation among life‐history groups observed here (Figure 3) complements other evidence which finds limited support for existing trait‐based descriptions of life‐history strategy as a predictor of community demographic variability in both corals and plants (Silvertown et al. 1992; Kuo et al. 2023; McWilliam et al. 2023). The 9‐year time series of community abundance at 15 distinct sites presented here provides a unique opportunity to evaluate existing notions of life‐history strategy and species function. For instance, it has been suggested that the *Pocillopora* dominated, and otherwise taxonomically limited, recovery of coral cover in Moorea and other locations in French Polynesia may not provide long term community stability (Berumen and Pratchett 2006; McWilliam et al. 2020; Pérez‐Rosales et al. 2021). Such views rely on *Pocillopora* being prone to population collapse as a result of its competitive life‐history strategy with associated high turnover rates, particularly for juvenile life stages (Darling et al. 2012; Kayal et al. 2015; Sarribouette et al. 2022). However, we found remarkable overall stability in *Pocillopora* spp. populations over the course of our study (Figures 1B and S3), which was second only to massive *Porites*, as has been found previously (Kayal et al. 2015). Similarly, some groups of massive corals, which are generally viewed as stress‐tolerant (Darling et al. 2012), experienced high variability and dramatic declines (e.g., massive species of *Pavona* and *G. stelligera*; Figures 1A and S3). On the other hand, as has been consistently found elsewhere, massive *Porites* was the most stable coral group, while encrusting groups such as *Montipora* and *P. superfusa*, and branching *Acropora* were unsurprisingly the most variable (Loya et al. 2001; Kayal et al. 2015; Moritz et al. 2018; Razak et al. 2019; Morais et al. 2021). Interestingly, a recent regional comparison of massive species of *Porites* and *Pocillopora* spp. showed remarkably similar median growth rates for these two key taxa, but different size‐frequency distributional characteristics (Sandin, Edwards, et al. 2020), further highlighting the need for comprehensive demographic studies of population change. Importantly, most taxa included in this study were highly variable in time and space (Figure S3), suggesting that context‐dependent effects are stronger drivers of population trends than putative life‐history strategies alone. Moreover, these results suggest that existing notions of life‐history strategy do not adequately describe the demographic capacity of corals, and their resulting patterns of intra‐ and interspecific variability.

The statistically‐driven stability observed in the first phase of this study suggests that taxonomic diversity, rather than functional diversity, should be an integral component of any model used to understand or predict change in high diversity coral communities. Further, results suggest that intense disturbance regimes can overwhelm the statistical effects of diversity and drive long term and widespread declines in population abundance and shifts in community structure. Moreover, the context‐specific variability (i.e., taxonomic, temporal and spatial) observed here underscores the need for more long‐term and well replicated demographic field studies of coral communities.

## Author Contributions

**Clinton B. Edwards:** conceptualization (lead), data curation (equal), formal analysis (lead), investigation (equal), validation (equal), writing – original draft (lead), writing – review and editing (equal). **Jonathan A. Charendoff:** investigation (supporting), methodology (supporting), writing – review and editing (supporting). **Vid Petrovic:** data curation (equal), methodology (equal), software (equal), visualization (equal), writing – review and editing (supporting). **Stuart A. Sandin:** conceptualization (supporting equal), formal analysis (supporting), funding acquisition (lead), investigation (equal), methodology (supporting), project administration (supporting), resources (equal), supervision (equal), writing – original draft (supporting), writing – review and editing (supporting).

## Funding

This work was supported by Beyster Family Foundation, Gordon and Betty Moore Foundation (3420).

## Conflicts of Interest

The authors declare no conflicts of interest.

## Supporting information


**Table S1:** COTS survey information.
**Table S2:** Observed coral taxa.
**Table S3:** Site‐level coral community abundance summary by study phase.
**Figure S1:** Map of Palmyra Atoll.
**Figure S2:** Site‐level focal coral abundance.
**Figure S3:** Focal coral abundance.
**Figure S4:** Functional group abundance (including non‐coral taxa).
**Figure S5:** Coral community evenness by site.

## Data Availability

The data that support the findings of this study are openly available on GitHub at https://github.com/clinton‐edwards.
