# Supplementary material for: Portfolio Effects and Synchrony in Coral Communities
Source: Ecol Evol. 2026 May 14;16(5):e73658. doi: 10.1002/ece3.73658 (PMC13175711; doi:10.1002/ece3.73658)

**Supplemental information for:**

**Portfolio effects and synchrony in coral communities**

Table of Contents

Table S1: COTS survey information 2

Table S2: Observed coral taxa 3

Table S3: Site level coral community abundance summary by study phase. 4

Figure S1: Map of Palmyra Atoll 5

Figure S2: Site-level focal coral abundance 5

Figure S3: Focal coral abundance 6

Figure S4: Functional group abundance (including non-coral taxa) 7

Figure S5: Coral community evenness by site 8

## **Table S1.** In 2017 opportunistic COTS abundance surveys were conducted at 18 sites around Palmyra. Surveys conducted within LAI plots included a 1m buffer around each plot (12 m x 12 m area surveyed). Additional surveys were conducted along the 10-m isobath with replicated 50 m x 3 m transects by teams of divers (1-3 divers, 1-9 total transects), with 1 m spacing between transects. Visual estimates of COTS predation were recorded as the proportion of affected colonies, recorded as follows: 0-25% (mild); 25-75 (heavy); and 75-100% (severe).

## **Table S2.** All 34 coral groups observed during the study. For groups comprised of >1 species we provide the list of species likely to occupy that group, as based upon the best available evidence and the expertise of the authors. Due to the difficulty of making species level designations with certainty from imagery, groupings were created conservatively. However, most groups with >1 species tended to comprised of 1-3 dominant taxa, as indicated here. Focal coral groups were identified as those whose island mean cover exceeded 0.5% during at least one year of the time series (notably, the same 12 groups of corals met a higher threshold of 1% island mean cover in at least one year). For each coral group identified in this study we provide the prevailing life-history classification as presented in Darling et al. (2012). There were no cases of disagreement in life-history classification for groups comprised of multiple taxa, though in several cases only a single taxon from the group had a known life-history classification (designated with a *). In some cases the life history classification was not known for individual taxa, and was instead inferred from similar species within the genus (designated with a **). Cases where no data was available to provide a reliable life-history classification are also identified (e.g., NDA).

| **Group** | **Primary taxon *(if applicable)*** | **Focal group** | **Life-history strategy** |
| --- | --- | --- | --- |
| ***Acropora (branching)*** *acuminata, muricata, robusta* | *muricata* | Y | Competitive* |
| ***Astrea curta*** |  | Y | Stress tolerant |
| ***Fungia*** *concinnna, fungites, granulosa, horrida, paumotensis, repanda, scutaria* | *concinna, paumotensis, scutaria* | Y | Stress tolerant* |
| ***Goniastrea stelligera*** |  | Y | Stress tolerant |
| ***Hydnophora microconos*** |  | Y | Stress tolerant |
| ***Montipora* (encrusting)** *capitata, efflorescens, flabellata, hemispherica, hoffmeisteri, informis, patula, tuberculosa, verilli, verrucosa* | inconclusive | Y | Generalist * |
| ***Pavona chiriquiensis*** |  | Y | Competitive |
| ***Pavona massive/submassive*** *duerdeni, explanulata, gigantea* | *duerdeni* | Y | Stress tolerant* |
| ***Pocillopora* spp.** *meandrina, verrucosa* | *meandrina, verrucosa* | Y | Competitive* |
| ***Porites* (massive)** *lobata, arnaudi, lutea* | *arnaudi* | Y | Stress tolerant* |
| ***Porites superfusa*** |  | Y | Weedy** |
| ***Turbinaria reniformis*** |  | Y | Competitive** |
| ***Acropora* (corymbose)** *cerealis, globiceps, nana, nasuta, secale, subulata, valida* | *nana, secale, valida* | N | Competitive* |
| ***Acropora* (tabular)** *clathrata, cytherea, hyacinthus* | *cytherea, hyacinthus* | N | Competitive* |
| ***Acropora*** *all species* |  | N | Competitive* |
| ***Astrea annuligera*** |  | N | NDA |
| ***Astreopora myriophthalma*** |  | N | Stress tolerant |
| ***Dipsastrea matthai*** |  | N | Stress tolerant |
| ***Favites* (encrusting)** *abdita, flexuosa, halicora, russelli* | inconclusive | N | Stress tolerant* |
| ***Favites pentagona*** |  | N | Stress tolerant |
| ***Gardineroseris planulata*** |  | N | Stress tolerant |
| ***Goniastrea pectinata*** |  | N | Stress tolerant* |
| ***Hydnophora exesa*** |  | N | Generalist |
| ***Leptastrea*** *purpurea, transversa, pruinosa* | *transversa* | N | Weedy |
| ***Leptoseris incrustans*** |  | N | NDA |
| ***Lobophyllia* spp.** *corymbosa, hemprichii* | *hemprichii* | N | Stress tolerant* |
| ***Montipora* (plating)** *aequituberculata, monasteriata, foveolata, caliculata* | inconclusive | N | Generalist * |
| ***Pavona varians*** |  | N | Stress tolerant |
| ***Platygyra* spp.***daedalea, pini, sinensis* | *sinensis* | N | Stress tolerant* |
| ***Pocillopora damicornis*** |  | N | Weedy |
| ***Pocillopora grandis*** |  | N | Competitive |
| ***Pocillopora sp.*** *meandrina, verrucosa, grandis* |  | N | Competitive* |
| ***Psamocora* spp.** *nierstaszi, profundacella* | *nierstrazi* | N | Generalist ** |
| ***Stylophora pistillata*** |  | N | Weedy |

## **Table S3.** Percent change (as a percentage of starting cover), mean and CV of total percent cover across each phase of the study by site.

## **Figure S1.** Map of Palmyra Atoll, a U.S. National Wildlife Refuge located approx. 1600km south of Hawaii. Since 2013 15 distributed along 10-m isobath of the northern and southern fore reef have been monitored annually using large-area imaging.


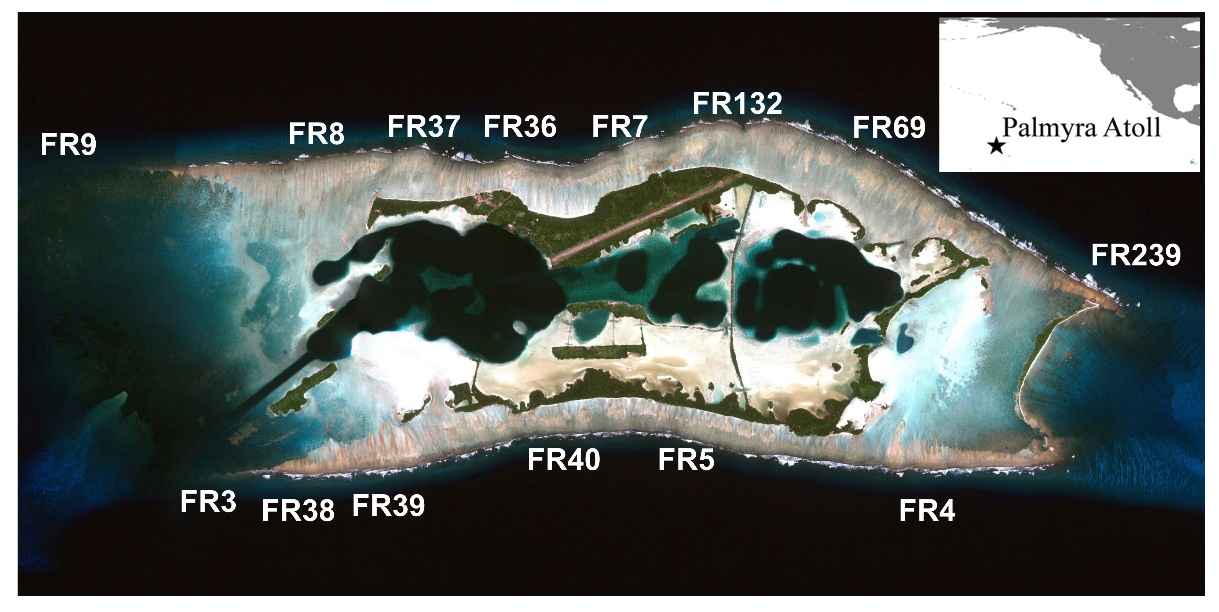


## **Figure S2.** Percent cover for each site by focal coral groups (lefthand axis) and total coral (righthand axis). Black vertical lines indicate the warm water associated coral bleaching event (2015) and the onset of the COTS outbreak (2017).


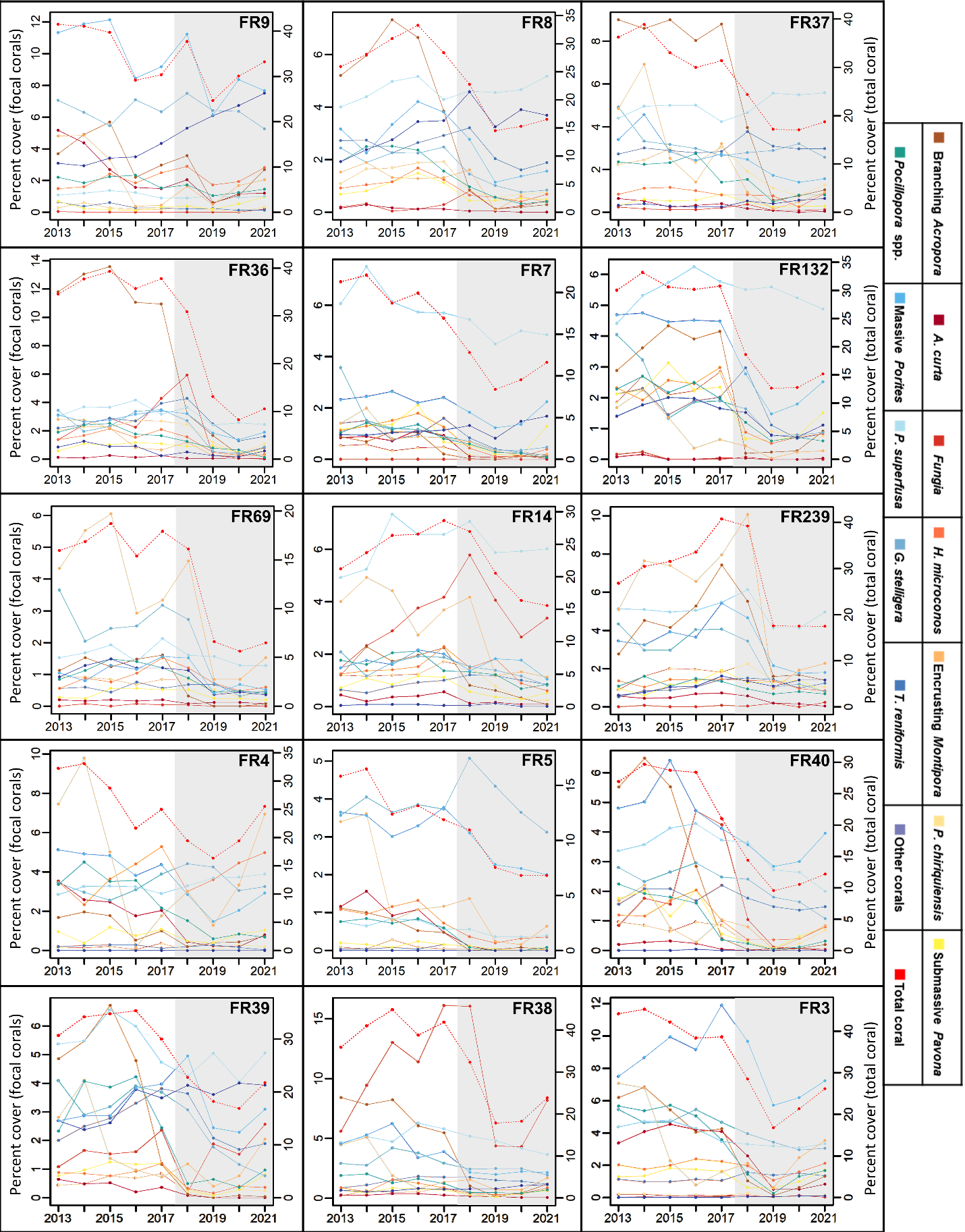


## **Figure S3.** Percent cover for each of the focal coral groups at each site. Black vertical lines indicate the warm water associated coral bleaching event (2015) and the onset of the COTS outbreak (2017).


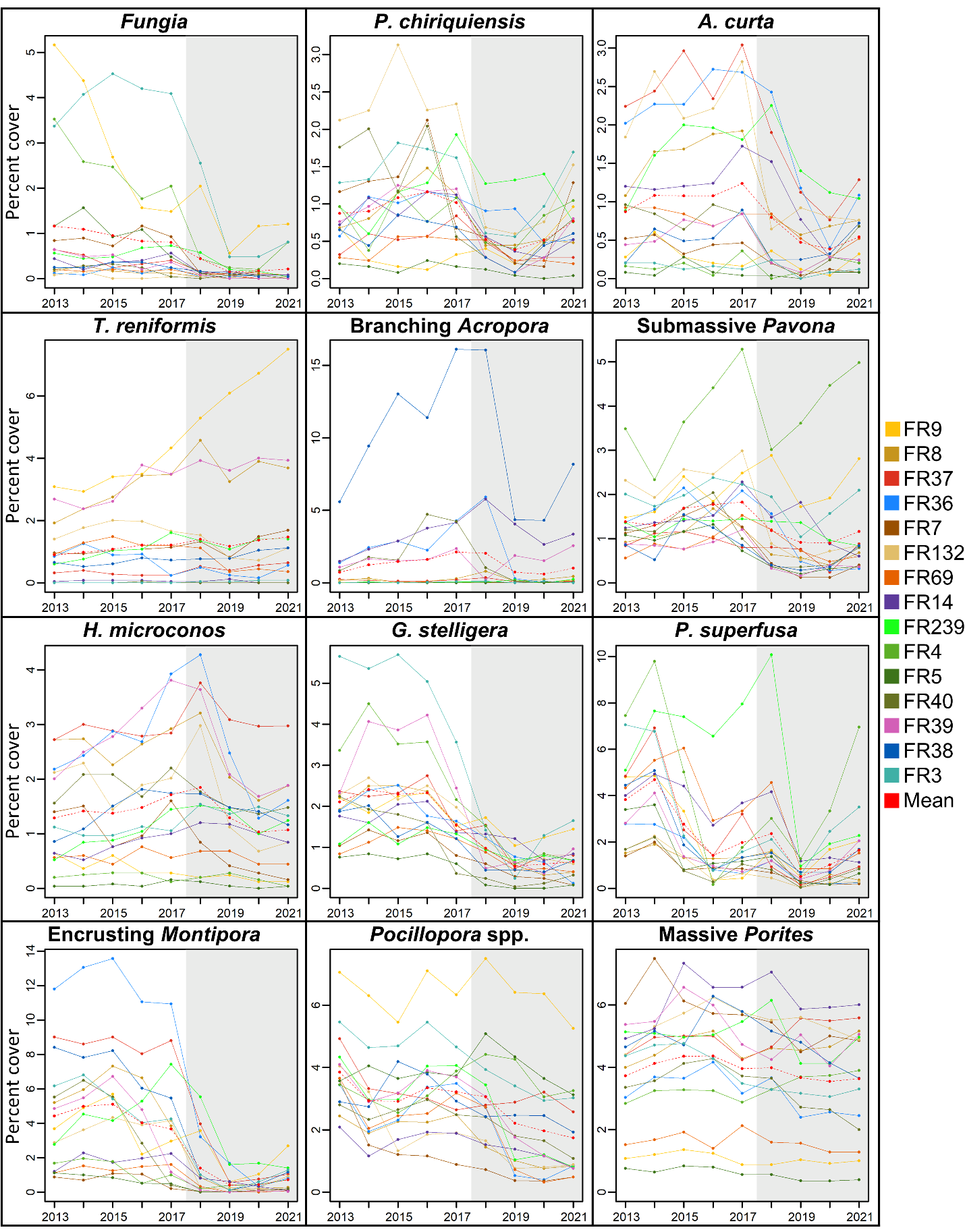


## **Figure S4**. Functional group percent cover for each site, and the island mean over the course of the study.


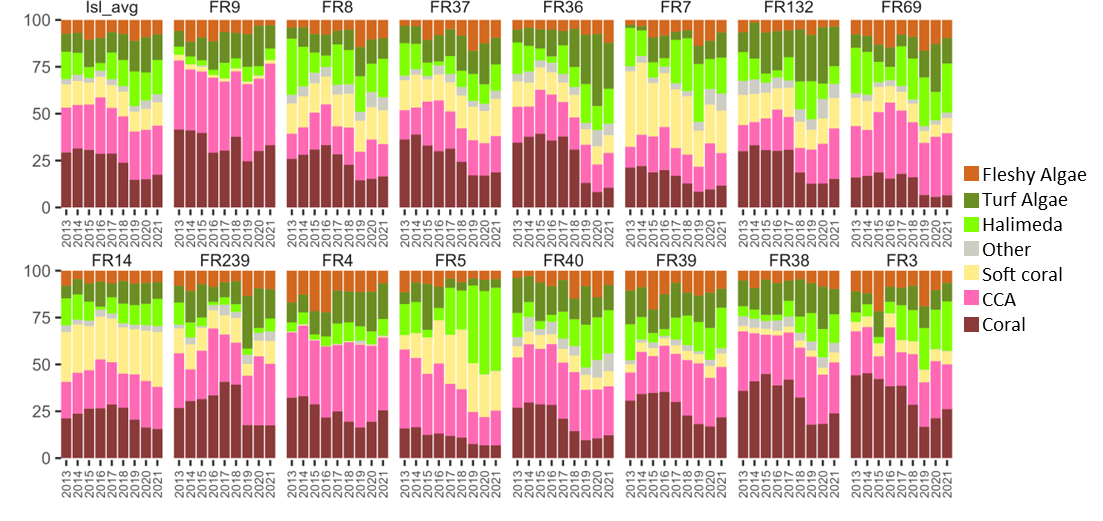


## **Figure S5.** Site level evenness through time. Evenness was calculated using the Simpson Index, $D=1/\sum_{i}^{S} p_{i}^{2}$, where S is the total number of species present in the community. D measures the probability that any two sampled individuals from a community will be of the same species and takes a maximum value of 1, corresponding to no diversity. Simpsons Index is commonly expressed as E=1/D, such that greater values correspond to higher levels of diversity and evenness.


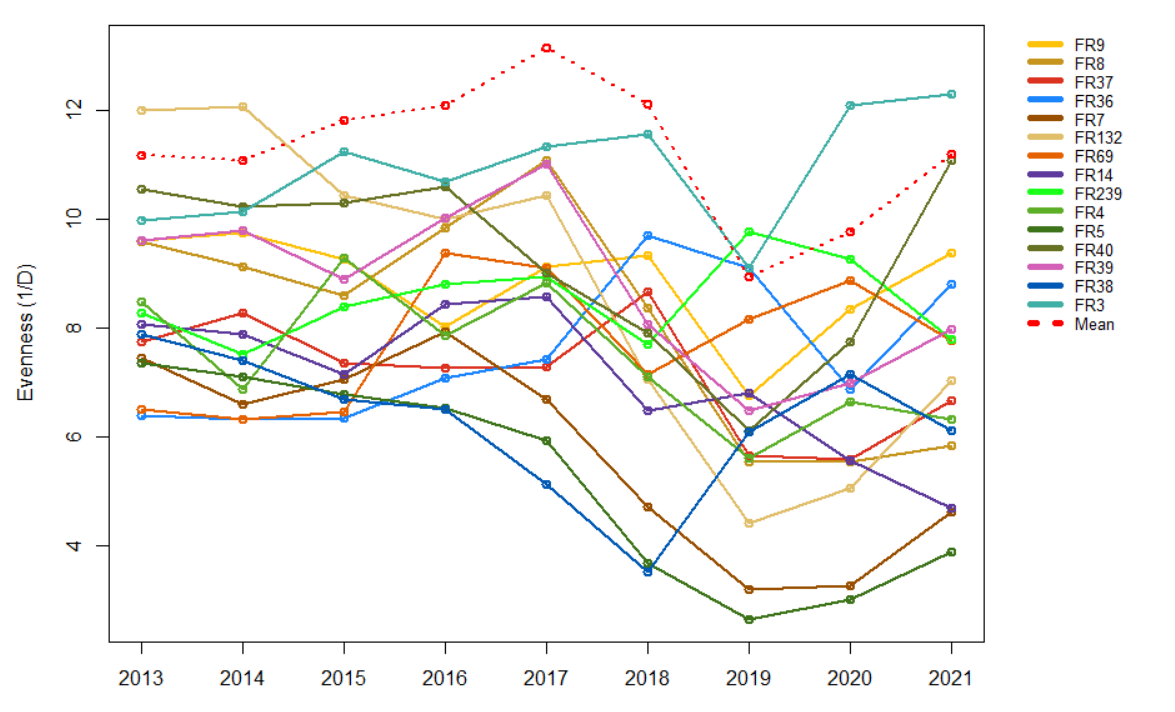

Supplement: Supplementary file 1 — Table S1: COTS survey information. Table S2: Observed coral taxa. Table S3: Site‐level coral community abundance summary by study phase. Figure S1: Map of Palmyra Atoll. Figure S2: Site‐level focal coral abundance. Figure S3: Focal coral abundance. Figure S4: Functional group abundance (including non‐coral taxa). Figure S5: Coral community evenness by site. [file ECE3-16-e73658-s001.docx]
